# Q fever in Bulgaria: Laboratory and epidemiological findings on human cases and outbreaks, 2011 to 2017

**DOI:** 10.2807/1560-7917.ES.2019.24.37.1900119

**Published:** 2019-09-12

**Authors:** Petia Genova-Kalou, Nadezhda Vladimirova, Savina Stoitsova, Stefka Krumova, Anna Kurchatova, Todor Kantardjiev

**Affiliations:** 1 National Centre of Infectious and Parasitic Diseases, Sofia, Bulgaria

**Keywords:** Q fever, outbreak, Bulgaria, laboratory study, epidemiological findings

## Abstract

**Background:**

Q fever is a zoonosis, included in category B of particularly dangerous infectious agents and as such merits careful surveillance and regular updating of the information about its distribution.

**Aim:**

This observational retrospective study aimed to provide an overview of Q fever incidence in Bulgaria in the period 2011 to 2017.

**Methods:**

Aggregated surveillance data from Bulgaria’s mandatory surveillance system, laboratory data on individual samples received at the National Reference Laboratory Rickettsiae and Cell Cultures and outbreak reports sent by the regional health authorities to the National Centre of Infectious and Parasitic Diseases, were used in this analysis. Cases were described by year, region, age group and most commonly identified risk behaviours.

**Results:**

A total of 139 confirmed cases were reported in the study period (average annual incidence: 0.27 cases/100,000 inhabitants). No seasonality or trend in reported cases was observed. Cases were mostly sporadic, with two small outbreaks in 2017. Identified risk behaviours among cases were occupational exposure and consumption of milk and dairy products, although exposure data were incomplete. The male/female ratio was 1.4. The identification and resolution of the two rural outbreaks in 2017 with a total of 18 cases involved good practices: active case finding and collaboration between public health and veterinary authorities.

**Conclusion:**

Between 2011 and 2017, Bulgaria retained low Q fever incidence, mostly sporadic cases and two small outbreaks. Occupational exposure and consumption of milk and dairy products were the most often reported likely exposures among cases. The outbreak investigations demonstrate the application of good control practices.

## Introduction

Q fever is an endemic zoonosis spread globally, except for New Zealand [1,2]. While the majority of cases are sporadic, several outbreaks among humans have been reported in different European countries (Germany, the Netherlands, Scotland and Slovenia) [3-6]. The aetiological agent of Q fever is the obligate intracellular bacterium *Coxiella burnetii,* included in category B of particularly dangerous infectious agents presenting a risk to human health, and it is considered as a potential weapon for bioterrorism [7]. The infectious agent has a wide range of animal hosts. [2,8]. In animals, infection is mainly subclinical but can also cause a range of conditions in livestock such as miscarriage, infertility, retained placenta, endometritis and mastitis. Infected animals shed large numbers of bacteria in placentas, vaginal discharge, faeces and urine [9,10]. Inhalation of pathogen-contaminated aerosol particles is the main route of infection in humans [11,12]. Consumption of unpasteurised milk also poses a risk, although it is considered lower [13]. The clinical presentation of Q fever in humans varies, ranging from asymptomatic infection, self-limiting febrile reaction, atypical pneumonia and acute or chronic granulomatous hepatitis to endocarditis in patients with pre-existing valvulopathy or vascular defects and meningoencephalitis in chronic disease forms [11,14-16]. Because the clinical presentation is similar to that of other diseases, Q fever often remains underdiagnosed [14-16].

In Bulgaria, Q fever in humans was first recognised by Mitov et al. in 1949 [17]. For more than 60 years, numerous sporadic cases and small and large epidemics, involving tens to hundreds of persons, occurred in different regions [18,19]. The last two major outbreaks in the country were registered in Etropole (2002) and in Botevgrad (2003–04) [20,21].

This study aimed to provide an overview of Q fever distribution in Bulgaria in the period 2011 to 2017, with consideration given to risk factors and possible underdiagnosis and underreporting.

## Methods

### Study design

A retrospective descriptive analysis of cases notified and reported in our mandatory surveillance system and of samples sent to the National Reference Laboratory for Rickettsiae and Cell Cultures (NRL RCC) was carried out. Cases and positive samples were described by region, age group, sex and year of notification/laboratory test. Data from outbreak reports, as received by the National Centre of Infectious and Parasitic Diseases (NCIPD), were described.

### Data sources and case definitions

In Bulgaria, Q fever is a mandatory notifiable disease and the European Union (EU) case definition and case classification have been used for surveillance purposes [22,23]. Epidemiological surveillance of human Q fever in Bulgaria is passive and aggregated. Cases are notified by primary reporting units (general practitioners, hospitals etc) to the Regional Health Inspectorates (RHI) of all 28 regions. The RHI then send aggregated reports on a weekly, monthly and annual basis to the National Center for Public Health and Analysis, which collates the data from all regions and forwards them to the NCIPD. Cases reported in monthly and annual reports are classified as probable or confirmed based on the EU case definition: A probable case is defined as any person meeting the clinical criteria (fever or pneumonia or hepatitis) with an epidemiological link, with epidemiological link defined as ‘at least one of the following two epidemiological links: (i) exposure to a common source, (ii) animal-to-human transmission’. A confirmed case is any person meeting the clinical and the laboratory criteria (*C. burnetii* isolation or detection of *C. burnetii* nucleic acid or *C. burnetii*-specific antibody response (IgG or IgM phase II)).

No information on distribution by risk factors is provided in the aggregated reports. Information on age group was routinely collected for the whole period 2011 to 2017, but information about sex distribution by age group has been collected only from 2014 onwards. Additionally, RHI send descriptive reports of identified outbreaks to the NCIPD. In this study, we analysed surveillance data on case numbers reported to NCIPD and case reports received at the NCIPD from 2011 to 2017.

Laboratory confirmation can be carried out in different laboratories. The NRL RCC at the NCIPD is the laboratory with the highest expertise in Q fever diagnosis in the country and receives a large number of samples for testing every year. The NRL RCC collects individual information on clinical presentation, demographics and risk behaviours, identified by physicians in communication with suspected cases and added to the information sent with the samples. However, confirmation by the NRL RCC is neither mandatory nor subsidised and therefore, sample sending practices to the NRL RCC differ by region. Some physicians and hospitals send samples primarily to the NRL RCC, while others choose other labs. In addition, not all cases are laboratory-confirmed. In this study, we analysed data on samples received at the NRL RCC between 2011 and 2017.

It must be noted that samples arrive at the NRL RCC with information regarding the location of the sending physician or hospital but without information on the residency of the patient. In this way, the regional distribution that can be derived from the sample information is not the same as the regional distribution of the cases reported through surveillance, as cases reported through surveillance are assigned to regions based on the residency of the patients.

Data on the regional population for each year under study was obtained from the official public database of the National Statistical Institute of Bulgaria [24].

### Laboratory methods

Serum samples from ambulatory and hospitalised patients with different clinical diagnoses were collected 1–3 weeks after the onset of clinical symptoms and sent to the NRL RCC. Two diagnostic methods (serology and/or molecular detection) were used. The human serum samples were tested for IgM phase II antibodies against *C. burnetii* with a commercial indirect enzyme-linked immunosorbent assay (ELISA) (SERION ELISA classic, *Coxiella burnetii* Phase II IgG/IgM, Virion/Serion, Würzburg, Germany), known to have high sensitivity (85%) and specificity (> 99%) [20].The assay was performed and interpreted as recommended by the manufacturer and the results were qualitatively categorised as positive, negative or equivocal. DNA was extracted from all IgM-positive human samples using the QIAamp DNA Blood Mini Kit (Qiagen Inc., Valencia, United States (US)). The extracted DNA was subjected to a conventional PCR assay (AmpliTaq Gold 360 DNA kit, ThermoFisher Scientifis, US) for the detection the *sodB* *C. burnetii* gene using the specific primers CB1 and CB2 [25]. All IgM-positive samples that were PCR-negative were further discussed, considering additional clinical and epidemiological information sent with the samples in the context of the timing of sample collection.

### Data analysis

The numbers of cases and annual incidence per 100,000 inhabitants reported through the surveillance source were described by year at the national level and by region for the whole study period. A test for trend over time was performed with year as independent variable and the annual number of reported cases as dependent variable in negative binomial regression, through which the incidence rate ratio (IRR), 95% confidence interval (CI) and p value were obtained. The available information from surveillance reports regarding two outbreaks in 2017 was also summarised.

The individual laboratory sample database from the NRL RCC was deduplicated. The number of sera tested and the number of samples positive in serology were summarised by year. The annual positivity rate (positive samples/total number of tested samples) was calculated. A test for trend over the years with regard to the number of positive samples and positivity rate was performed at the national level, using negative binomial regression to calculate IRR and 95% CI. A test for seasonality in confirmed samples was also performed through calculating the correlation between the detrended series and sine curves in order to establish whether there was a dominant periodicity in the time series. We used the Wilcoxon rank-sum test to test the hypothesis that April to October is a period with more positive samples and the hypothesis that June to July is a peak period. Samples sent per 100,000 inhabitants and positivity rate by region were compared. The distribution of positive samples by sex, age, clinical symptoms and suspected risk factors was summarised.

### Ethical statement

We present surveillance data collected routinely by the national reference laboratory and surveillance units in the country. Data are presented in aggregated and anonymous format. Publication of this analysis does not harm or influence neither cases nor institutions. Ethical committee approval was therefore not required.

## Results

### Overall numbers and temporal distribution of cases

In the period from 2011 to 2017, a total of 148 cases of Q fever in humans were reported through the national surveillance system, of which 139 were classified as confirmed based on the EU case definition. The cases, mostly sporadic, were reported by 18 of the 28 regions in the country. In 2017, two limited outbreaks occurred, with fewer than 10 cases each. The average annual incidence of Q fever at the national level was 0.29 per 100,000 inhabitants (ranging from 0.16 (2011) to 0.42 (2017)) (Table). There was no statistically significant trend or seasonality observed in the annual national incidence as calculated from surveillance data.

**Table:** Annual laboratory and surveillance data regarding samples tested for *Coxiella burnetii* at the NRL RCC and confirmed cases reported through the surveillance system, Bulgaria, 2011–2017 (n = 1,430)

Year	Laboratory database			Surveillance data			
	Number of sera tested	Number of positive samples (IgM phase II)	Positivity rate (%) *C. burnetii*-positive human samples	Total notified cases	Notification rate (cases/ 100,000 population)	Confirmed cases (EU case definition)	Incidence rate (confirmed cases/100,000 population)
2011	146	12	8.2	12	0.16	12	0.16
2012	96	9	9.4	29	0.40	29	0.40
2013	169	15	8.9	23	0.32	23	0.32
2014	189	26	13.8	17	0.23	15	0.21
2015	228	28	12.3	18	0.25	15	0.21
2016	229	33	14.44	19	0.27	17	0.24
2017	373	38	10.2	30	0.42	28	0.39
**Total**	**1,430**	**161**	**11.3**	**148**	**0.29^a^**	**139**	**0.27**

EU: European Union*;* NRL RCC: National Reference Laboratory Rickettsiae and Cell Cultures.

^a^ Average annual notification rate for the period.

For the same period (2011–17), a total of 1,430 serum samples from patients with suspected Q fever were received at the NRL RCC from 15 regions. By means of IgM phase II ELISA, antibodies against *C. burnetii* were detected in 161 (11.3%) of the tested human sera. Sera from all IgM-positive patients were additionally tested by conventional PCR assay and the presence of *C. burnetii* DNA was confirmed in 143 samples (88.8%). After taking into account clinical and epidemiological information of the IgM-positive samples that were PCR-negative and the timing of sample collection, all IgM-positive samples were finally classified as laboratory-confirmed and are presented here (Table). A very small significant positive trend at the national level was detected for the annual number of samples received by the laboratories (IRR = 1.01; 95% CI: 1.01–1.02) and for the number of IgM-positive samples (IRR = 1.02; 95% CI: 1.01–1.03) but not for the positivity rate. There was no seasonality in the data (data not shown). Nor was there a significant difference between the periods April to October and November to March and between the periods June to July and August to May with regard to monthly number of tested samples, number of positive samples and positivity ratio.

### Regional distribution of cases and positive samples

**Figure 1:**
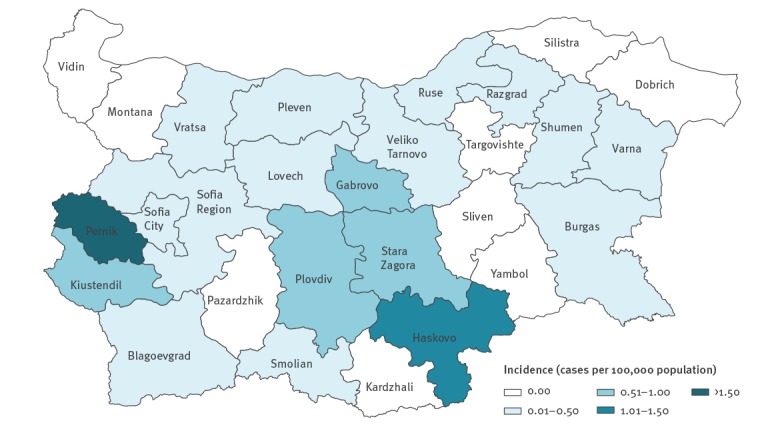
Average annual regional incidence of confirmed Q fever cases reported through surveillance, Bulgaria, 2011–2017 (n = 1,430)

Average annual regional incidence rates of confirmed Q fever cases, as derived from surveillance data, are presented in Figure 1. Nine of 28 regions did not notify any cases during the study period. Twelve regions had an average annual incidence for the period below 0.5 per 100,000 inhabitants. Six regions stood out with higher than average incidence: Pernik, Haskovo, Stara Zagora, Kiustendil, Gabrovo and Plovdiv with average annual incidences of, respectively, 2.81, 1.12, 1.00, 0.98, 0.76 and 0.65 cases per 100,000 inhabitants (Figure 1). It must be noted that among those regions, Pernik, Gabrovo and Kiustendil differed from the other regions in their practices for sending samples to the NRL. Per 100,000 inhabitants, Pernik and Gabrovo sent the highest number of samples to the NRL RCC, with Pernik sending 10 times more than the average and Gabrovo sending four times more than the average (data not shown).

### Age and sex distribution of IgM-positive samples tested at the NRL RCC

**Figure 2:**
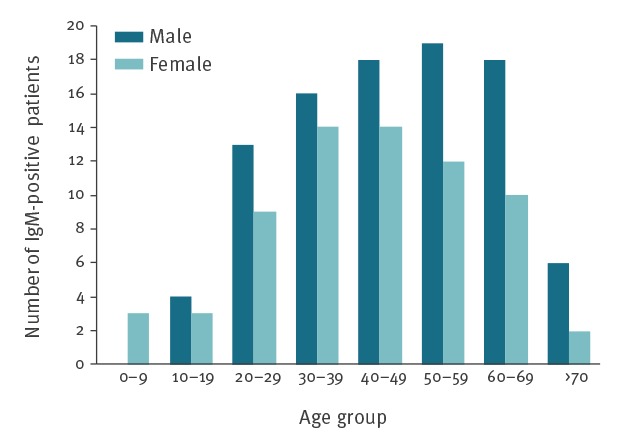
Human *Coxiella burnetii* IgM phase II-positive samples, by age group and sex, Bulgaria, 2011–2017 (n = 161)

The median age of people with positive samples was 45 years (age range: 2–78). The smallest numbers of patients with positive samples were observed among children younger than 10 years and people older than 70 years. Of the 161 laboratory-confirmed samples, 94 (58.4%) were from male patients and 67 (41.6%) were from female patients (Figure 2). Overall, the number of male patients with positive samples was significantly higher than the number of female patients with positive samples for Q fever (chi-squared p<0.05). The male to female ratio was 1.4.

### Q fever-positive patients by clinical diagnosis

Within the study period, 74 of the 161 laboratory-confirmed Q fever cases were initially diagnosed with fever of unknown origin. Another 72 confirmed patients were hospitalised with atypical pneumonia and one was diagnosed as acute bronchitis. The remaining IgM phase II-positive cases were hospitalised with heart disease diagnoses: nine with endocarditis, three with pericarditis and two with myocarditis.

### Possible occupational and other risk factors for Q fever

Limited epidemiological information was available in the laboratory database, where risk behaviours (i.e. suspected exposures) were identified for only 58 (36.0%) of IgM phase II-positive patients. Among the 58 for whom risk behaviour information was available, occupational hazards were identified for 25, while for 33, consumption of milk and dairy products from private stockbreeders was accepted as the possible risk for Q fever infection.

### Q fever outbreak in the region of Blagoevgrad, 2017

On 13 March 2017, a resident of a village in Blagoevgrad region called the RHI and reported about an exceptionally large number of people living in the village who were suffering from pneumonia. On the next day, an investigation was initiated. The RHI began a retrospective search in the local hospitals for patients living in the same village and hospitalised with acute respiratory findings within the previous 2 months. The population of the affected village is about 750 people. A total of 35 were identified to have been hospitalised with pneumonia between 22 February and 10 April. Samples were taken from 32 of them. Eleven of the 32 samples were positive for C*. burnetii* (IgM phase II positivity). The age range of positive cases was 18–55 years and the median age was 32 years; six were women and five were men. The cases’ places of residence were scattered throughout the village. Sometimes more than one case was diagnosed in a family. Six cases gave information about consumption of milk from domestic goats while the rest shared information about exposure to the local goat herd only on the way of the animals to the pasture. The RHI contacted the regional veterinary centre and asked for investigation of private ruminants (sheep and goats) in the village. Q fever-positive goats were identified by the veterinarians and it was concluded that the source of this outbreak had been domestic goats. The possible spread was through infected aerosol and/or infected milk (in some households).

### Q fever outbreak in the region of Gabrovo, 2017

A routine serological screening among sheep and goats, carried out in the Gabrovo region, established *C. burnetii* positivity in sheep and goats from one village. After the signal from the veterinarians, an epidemiological investigation was conducted in mid-November 2017 among workers in sheep breeding facilities in the area. A total of 39 samples were collected of which seven were positive for *C. burnetii* (IgM and PCR positivity). The age range of positive workers was 40–68 years and the median age was 50 years; five were women and two were men. Five reported having had fever of unknown origin and two had atypical pneumonia, but none had been hospitalised.

### Control measures

Q fever control measures in Bulgaria according to standard guidelines for veterinary [26] and public health [27,28] authorities include: (i) hospitalisation and appropriate treatment of patients with antibiotics (tetracyclines, quinolones or macrolides in effective doses for at least 2–3 weeks), (ii) follow up of contacts for 30 days, including two serological tests for *C. burnetii* per contact within this period, (iii) disinfection of the environment and work place, (iv) manure composting for at least 6 months under nylon or treatment of manure with lime, (v) temporary halting of milk collection from animals positive for Q fever, lasting until the animals had completed antibiotic treatment (vi) pasteurisation of milk, (vii) measures related to animals, as implemented by veterinary authorities, including establishing a separate birthing zone, removal of placenta and disinfection of the birthing zone after birthing and active animal case finding within the affected herd, (viii) enhanced surveillance during the outbreak (contact tracing and active case finding), (ix) health promotion activities, including the distribution of information, reinforcing messages about good preventive practices in livestock farming (separate birthing areas, regular disinfection, personal protective equipment such as gloves, gumboots, protective clothing, eye protection and respiratory protection, cleaning, handwashing, etc.)

In the case of the two outbreaks, the RHI reported having applied all appropriate measures. Active case finding and collaboration with the veterinary authorities in both cases contributed to better characterisation of the outbreaks. It must be noted that because of the complexity of the diagnosis, both outbreaks were detected at a late stage and therefore, not all measures could be implemented in the most timely manner. Nevertheless, information about the findings was immediately shared by the regional health authorities with various stakeholders (veterinary authorities, representatives of the respective municipalities etc.), and field work related to enhancing control measures was carried out in order to raise awareness about prevention and control measures and prevent future outbreaks.

## Discussion

Most Q fever cases registered in Bulgaria between 2011 and 2017 were sporadic, similar to other EU countries [29]. Notification rates were low for the whole period, with the average notification rate comparable to the average EU levels observed in 2014, 2015 and 2016 (average notification rate for EU for this period was 0.2 cases per 100,000 population) [29]. Q fever cases are registered during the entire year in Bulgaria without significant seasonality in reporting. Overall in the countries in the EU and European Economic Area (EEA), cases are also reported throughout the year. However, combining the data from the all EU/EEA countries leads to an observable seasonality, with peaks in June and July [30]. Interestingly, European surveillance data from 2016, unlike data from other years, show no clear seasonality in the EU/EEA, because France and Germany reported consistently higher case numbers from January to August 2016 [29]. The lack of statistically significant seasonality in Bulgaria may be due to the overall small number of notified cases, to differences in farming practice or to other reasons. The data available to us do not allow us to draw definitive conclusions with regard to the determinants behind the observed lack of seasonality. Some regions are affected more than others, which may be due to the specific economic activities in these regions, but could also be attributable to varied detection of cases, considering that the diagnosis is complex and symptomatic cases may remain undiagnosed because of non-specific symptoms, as reported elsewhere [31]. It is indeed possible that the differences in notification rates among regions are due to a combination between variation in risk factors and variation in surveillance and testing practices. Information on risk behaviours among cases was incomplete. Nevertheless, it helped identify the most important suspected risk behaviours such as occupational hazard and consumption of milk and dairy products. While this information is indicative of likely exposure, lack of information on the prevalence of the same behaviours among a control group limits our ability to establish which exposures are truly risk factors. There were more cases among men, which may be linked to their higher likelihood of occupational exposure – a hypothesis that needs to be confirmed further through dedicated studies. 

The non-specific clinical symptoms make it difficult to diagnose Q fever. Therefore, laboratory diagnostic capacities should allow for quick identification, but also for the differentiation between acute and past Q fever infections. This is why the NRL RCC employs both serological and PCR testing in order to improve the diagnostic capacities with regard to this disease.

Over the studied period, we observed a small significant positive trend for number of tested samples and number of positive samples, but not for the positivity rate. This may indicate improvement in diagnostic practices (i.e. sample sending to the NRL RCC) rather than an increase in incidence. There are indications of underreporting based on our data, especially for the last years, as there are more positive samples from the NRL RCC than reported cases through surveillance. Unfortunately, there is no means for us to link the individual dataset from the NRL RCC and the aggregated surveillance data in order to pinpoint the reasons for the difference between the two systems. This issue demonstrates the importance of introducing case-based surveillance integrating laboratory and surveillance data in order to monitor and improve surveillance performance. An additional limitation of our study is that the ELISA test used has a sensitivity of 85%, which can lead to false negative results and an underestimation of positivity rates. For diagnostic purposes, the NRL RCC tested by PCR some IgM-negative samples for which laboratory material was available and samples that were considered equivocal (data not shown). However, resource limitations did not allow this to be done in a systematic manner, and in order to present and analyse systematically collected data, we have summarised here only the data for IgM-positive samples, for which the laboratory results had been aligned with the clinical manifestation of Q fever in patients.

In 2017, in addition to sporadic reports of Q fever cases, two limited outbreaks were reported by the local hospitals and the RHI in two regions in Bulgaria. All cases were among rural populations; one was suspected to be linked to consumption of milk and dairy products and the other to occupational exposure. Both outbreaks involved active case finding and demonstrated how this approach leads to identification of more cases, which underlines the importance of active case finding during outbreaks. In addition, the outbreaks were good examples of collaboration between public health and veterinary units in the control of zoonotic diseases.

### Conclusions

During the period from 2011 to 2017, Bulgaria retained a low Q fever incidence with mostly sporadic cases and two small outbreaks. Occupational exposure, and consumption of milk and dairy products were the main risk factors, and men were more affected by the disease. The NRL RCC employs methods that allow quick diagnosis of cases and differentiation between current and past infections and is best placed to provide accurate and experienced diagnosis of suspected cases. The practices of sending samples to the NRL RCC have improved during the studied period – a positive development that should continue in the future. At the same time, our data indicate underreporting, which could be addressed by introducing case-based surveillance, although this is strongly dependent on available resources. The outbreak investigations carried out in 2017 involved active case finding and collaboration with veterinary units and demonstrate the application of good practices to limit the spread of Q fever.
